# Numerical modeling of nanodrug distribution in tumors with heterogeneous vasculature

**DOI:** 10.1371/journal.pone.0189802

**Published:** 2017-12-29

**Authors:** Cheng-Ying Chou, Wan-I Chang, Tzyy-Leng Horng, Win-Li Lin

**Affiliations:** 1 Department of Bio-Industrial Mechatronics Engineering, National Taiwan University, Taipei, Taiwan; 2 Department of Applied Mathematics, Feng Chia University, Taichung, Taiwan; 3 Institute of Biomedical Engineering, National Taiwan University, Taipei, Taiwan; 4 Institute of Biomedical Engineering and Nanomedicine, National Health Research Institutes, Zhunan Township, Miaoli County, Taiwan; Academia Sinica, TAIWAN

## Abstract

The distribution and accumulation of nanoparticle dosage in a tumor are important in evaluating the effectiveness of cancer treatment. The cell survival rate can quantify the therapeutic effect, and the survival rates after multiple treatments are helpful to evaluate the efficacy of a chemotherapy plan. We developed a mathematical tumor model based on the governing equations describing the fluid flow and particle transport to investigate the drug transportation in a tumor and computed the resulting cumulative concentrations. The cell survival rate was calculated based on the cumulative concentration. The model was applied to a subcutaneous tumor with heterogeneous vascular distributions. Various sized dextrans and doxorubicin were respectively chosen as the nanodrug carrier and the traditional chemotherapeutic agent for comparison. The results showed that: 1) the largest nanoparticle drug in the current simulations yielded the highest cumulative concentration in the well vascular region, but second lowest in the surrounding normal tissues, which implies it has the best therapeutic effect to tumor and at the same time little harmful to normal tissue; 2) on the contrary, molecular chemotherapeutic agent produced the second lowest cumulative concentration in the well vascular tumor region, but highest in the surrounding normal tissue; 3) all drugs have very small cumulative concentrations in the tumor necrotic region, where drug transport is solely through diffusion. This might mean that it is hard to kill tumor stem cells hiding in it. The current model indicated that the effectiveness of the anti-tumor drug delivery was determined by the interplay of the vascular density and nanoparticle size, which governs the drug transport properties. The use of nanoparticles as anti-tumor drug carriers is generally a better choice than molecular chemotherapeutic agent because of its high treatment efficiency on tumor cells and less damage to normal tissues.

## Introduction

Nanodrug carriers are advantageous over conventional molecular medicine in cancer therapy due to their higher tumor selectivity [1]. The therapeutic efficiency of anti-cancer drugs is highly correlated with their spatial and temporal concentration distributions in the tumor, which are governed by the tumor environment [2] and the physicochemical properties of a drug. The uniformity of the drug concentration distribution affects the therapeutic effect on the entire tumor, and the cumulative concentration dominates the survival rate of cells. Therefore, the aim of drug delivery is to achieve a high and uniform distribution of the cumulative drug concentration in a tumor. Since drug delivery relies on the vascular system, an abnormal vasculature affects the deposition of drug molecules in a tumor through blood vessels. The presence of the high interstitial pressure in the tumor also hinders the drug delivery [3, 4]. The drug molecules are extravasated from blood vessels, and their transport in the interstitium is driven by diffusion and convection effects. Diffusion effect is caused by the concentration difference in the interstitium, while the convection effect is driven by the interstitial pressure gradient.

**Fig 1:**
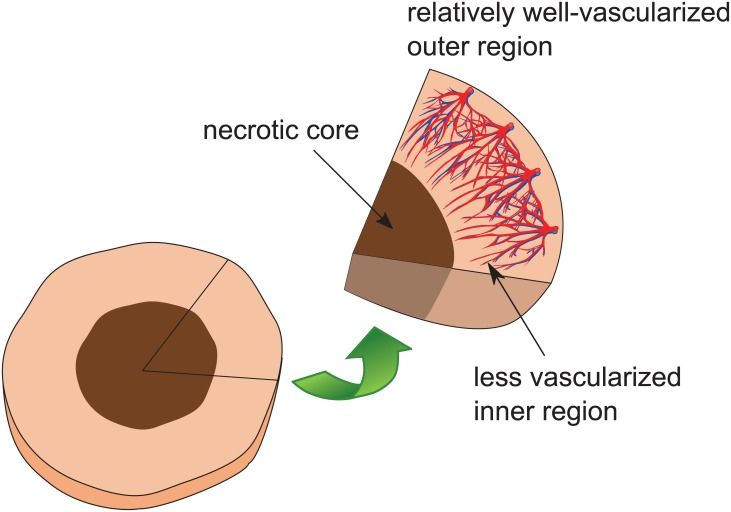
The cross-section illustration of a tumor with a necrotic core.

The concentration difference in the interstitium is mainly the result of the heterogeneous vascular distribution in the tumor [5]. Tumor blood vessels are highly irregular in their structure compared with those in normal tissues. Unlike normal vessels, tumor vessels are dilated and tortuous, and their vascular walls are leaky and more permeable than normal vessels [6–8]. Moreover, the vascular distribution of tumor is highly heterogeneous. Tumor angiogenesis starts from the outer region and then spreads into the inner region. The proliferation of tumor cells results in a well-vascularized region in the periphery and a less vascularized region near the tumor center, in which a necrotic core may form, as illustrated by Fig 1. The heterogeneity of the blood vessel network leads to a non-uniformly cumulative concentration distribution of the drug within the tumor. In the tumor, the interstitial pressure is high and the interstitial pressure gradient is near zero due to a less functional lymphatic network. The function of a lymphatic network is to drain excess fluid from tissues to maintain the interstitial fluid balance and to prevent the occurrence of high pressure. However, functional lymphatic vessels can only be found in the tumor periphery, and the lymphatic vessels together with blood vessels at the center of a tumor are compressed by cancer cells and therefore often collapsed [9, 10]. As mentioned in the previous paragraph, the tumor vessel walls are leaky and thus fluid can easily leak from blood vessels to tumor tissues. The less functional lymphatic system in a tumor gives rise to the insufficient drainage of fluid, thereby leading to the fluid accumulation in the interstitium and a high interstitial pressure around the center of tumor tissues. On the other hand, the vasculature at the outer region of a tumor can drain the excessive fluid; therefore, the interstitial pressure drops quickly [9, 10]. The pressure gradient at the periphery region induces an outward convection, which pushes drug particles away from the tumor.

The tumor cell survival rate can serve as an indicator to evaluate the therapeutic effect and to estimate the probability of tumor recurrence. Putten and Lelieveld reported that there existed a log-linear relationship between the tumor survival rate and the extracellular drug concentration [11]. However, El-Kareh and Secomb also argued that the cell survival rate was more closely related to the intracellular drug concentration than the extracellular concentration [12]. Nevertheless, both works suggested that the tumor survival rate was proportional to the drug concentration whether it is intracellular or extracellular. High drug concentrations generally lead to a lower survival rate as more cells are killed by the drug. In this work, we developed a mathematical tumor model starting from the governing equation describing interstitial pressure distribution introduced by Soltani and Chen [13], and in addition we added the drug transport model to depict the temporal evolution and spatial distribution of drug concentration. The model was applied here to study a subcutaneous tumor with heterogeneous vascular networks considering the physiological characteristics of a tumor. Both nanoparticle carriers and a molecular therapeutic agent were investigated. Their difference in terms of the therapeutic effect and damage to the surrounding normal tissues were compared. The objective is to investigate the influence of tumor characteristics and drug properties on the drug AUC (area under the curve) distributions in the entire tumor by numerical simulation of the proposed mathematical model. In addition, the cell survival rate was estimated to quantify the therapeutic effect.

Since this work is primarily focused on drug delivery, we did not study the detailed tumor growth model, but described it by a simplified equation (Eq (23)). Because little is known about the regrowth of human tumors after chemotherapy [14], several methods have been proposed to model the kinetics of tumor repopulation [15–19]. One of the most popular models is to assume an exponential growth of tumor cells while accounting for a decreased growth rate as the volume of tumor increases [17]. The tumor growth is considered to follow the Gompertz model, which has become a widely accepted growth process for tumor growth in particular [18, 20, 21]. Consequently, we employed the Gompertz model to simulate the tumor repopulation after chemotherapy.

## Materials and methods

The anti-cancer drug concentration in a tumor is affected by the vascular density, the size of a tumor, and characteristics of a drug such as molecular weight, diffusivity, vascular permeability, uptake rate by tumor cells, and the plasma half-life. Doxorubicin and various sized dextrans were considered and they were assumed to be well circulated in the body. The drug concentration in the plasma will gradually diminish due to the drainage by the lymphatic system and tissue absorption, as well as the clearance by the body.

### Geometric configuration

In our model, tumors were assumed to be spherical and symmetrical. Unlike previous studies in Baxter and Jain [22], the vascular distribution of the tumor was modeled to be spatially heterogeneous and the vascular system comprised arterial capillaries and lymphatic vessels.

The value of the current one-dimensional (1D) model relies on its consistent and detailed governing equations for drug transport. In spite of simplification in geometry compared with two, three-dimensional (2D/3D) models [23–25], a well-constructed 1D model still can explore important physical mechanisms of drug transportation in tumors, and offer significant tumor treatment advices. A good 3D model sure can do better in prediction, but it takes long computing time and huge amount of resources to run a case, as compared to 1D models. Especially, numerous numerical simulations are required for testing and fitting the model parameters and analyzing their sensitivity afterwards, which will be an exhaustive task for 3D models, but they are much easier to be implemented in 1D models. 1D models do have their limitations compared with 2D/3D models. Usually a good tumor mathematical model is hierarchical with dimensions, and 1D model can provide a good test of mathematical model and identify important parameters efficiently before extending to 2D/3D.

Both an isolated and a subcutaneous tumors can be investigated by this model. The subcutaneous tumor is surrounded by normal tissues while an isolated tumor is not. A tumor region can be divided into a vascularized and necrotic regions. The vascularized region is mostly in the peripheral region of a tumor while the necrotic region comprises the tumor core. Though the proposed model is versatile, only the case of a subcutaneous tumor is presented in the current work.

**Fig 2:**
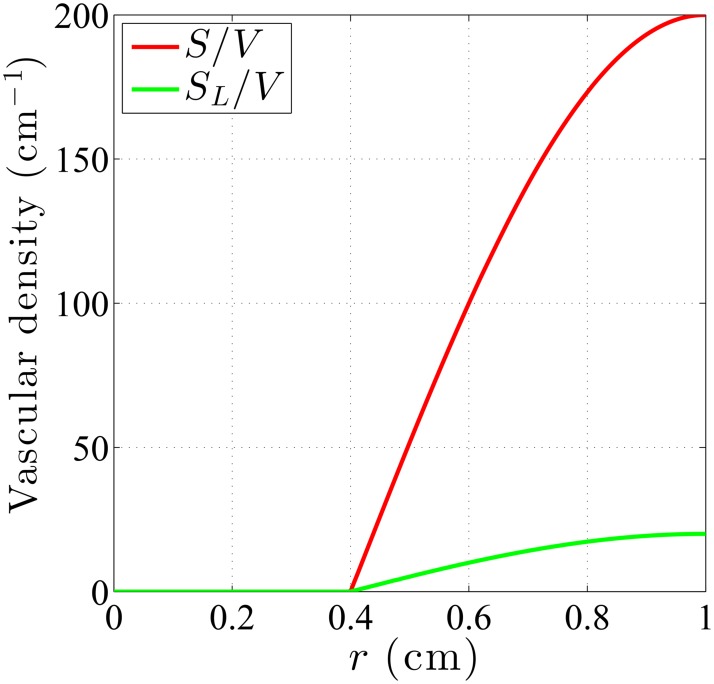
A sine function was used to model the tumor vascular distribution. Vascular density distribution in a tumor with a necrotic core. The sine function exists only outside the necrotic region and is highest at the tumor boundary. Vascular density is zero throughout the necrotic core.

One of the key factors needs to be considered in tumor modeling is the vasculature in tumor. The vasculature of tumors, featuring heterogeneous distribution of vessel sizes and shapes in 3D computations, is simplified here by the vascular density distribution *S*/*V*, i.e., the surface area of blood vessel (*S*) per unit volume of tumor (*V*). The vascular density is assumed to be zero in the necrotic core and then increase radially from the necrotic core boundary, reaching highest at the tumor boundary since the vascularized region is mostly in the peripheral region. A typical vascular density distribution in a tumor with a necrotic core is illustrated in Fig 2 in which the radius of the necrotic core is set to *R*_*n*_ = 0.4*R* [8], where *R*_*n*_ and *R* denote the radius of the necrotic core and the tumor, respectively. For normal tissues, vascular density distribution was assumed to be homogeneous and therefore a constant value for *S*/*V*, since the vasculature in normal tissues is generally well structured. As vasculature consists of arterial and lymphatic(venous) capillaries, both the arterial and lymphatic vascular density distributions of the tumor, *S*/*V* and *S*_*L*_/*V*, are assumed to be zero in necrotic core and otherwise a sine function here, as shown in Fig 2. Venous vessels have similar functions as lymphatic vessels; therefore, the presence of venous vessels will be omitted in the paragraphs that follow. A major difference between the current work and Baxter and Jain [22] is that they considered *S*/*V* distribution to be uniform in both tumor and normal tissues under different constant values, and their vasculature only consisted of arterial capillaries without considering the drainage by venous/lymphatic capillaries.

### Interstitial pressure

To reach targeted tumor cells, systemically delivered drug needs to first extravasate from blood vessels, passes through the extracellular matrix, and finally penetrates into intracellular sites. The extravascular transport of drug carriers relies on convection and diffusion, which are determined by the velocity of the interstitial fluid and the drug concentration distribution. The fluid velocity is proportional to the interstitial pressure gradient as described by Darcy’s law,

$$\begin{array}{c} \vec{u}=-k\nabla P_{i}, \end{array}$$ (1)

where *k* (cm^2^/mmHg-s) is the hydraulic conductivity of the interstitium and *P*_*i*_ (mmHg) is the interstitial pressure. In the biological tissues with the fluid source and fluid sink in the medium, the continuity equation for the steady-state incompressible flow should be modified as $\nabla \cdot \vec{u}=\phi_{B}+\phi_{L}$, where *ϕ*_*B*_ and *ϕ*_*L*_ (s^-1^) are the fluid source from blood vessels and the sink by lymphatic drainage, respectively. The fluid source term denotes the flow flux out of the vascular wall per unit volume, and is governed by Starling’s law as follows:

$$\begin{array}{c} \phi_{B}=\frac{L_{p}S}{V}[P_{B}-P_{i}-\sigma_{s}\left(\pi_{B}-\pi_{i}\right)], \end{array}$$ (2)

where *ϕ*_*B*_ is the volumetric flow rate out of blood vessels per unit volume of the tumor, *L*_*p*_ is the hydraulic conductivity of the vascular wall (cm/mmHg-s); the vascular density *S*/*V* is the blood vessel surface area per unit volume of the tumor (cm^-1^); *σ*_*s*_ is the average osmotic reflection coefficient; *P*_*B*_ and *P*_*i*_ are pressure in blood vessels and interstitium (mmHg); *π*_*B*_ and *π*_*i*_ are the osmotic pressure of the plasma and interstitial fluid (mmHg).

The lymphatic drainage term is described similar to blood vessel, but without osmotic pressure difference:

$$\begin{array}{c} \phi_{L}=\frac{L_{pL}S_{L}}{V}\left(P_{L}-P_{i}\right), \end{array}$$ (3)

where −*ϕ*_*L*_ is the volumetric flow rate into lymphatic vessels per unit volume of the tumor, *L*_*pL*_ is the hydraulic conductivity of lymphatic wall (cm/mmHg-s), the lymphatic vascular density *S*_*L*_/*V* is the lymphatic vessel surface area per unit volume of tumor (cm^-1^), and *P*_*L*_ is the pressure in lymphatic vessels (mmHg).

Combining Darcy’s Law and the continuity equation with the substitution of *ϕ*_*B*_ and *ϕ*_*L*_ can result in

$$\begin{array}{c} -k\nabla^{2}P_{i}=\frac{L_{p}S}{V}\left[P_{B}-P_{i}-\sigma_{s}\left(\pi_{B}-\pi_{i}\right)\right]-\frac{L_{pL}S_{L}}{V}\left(P_{i}-P_{L}\right). \end{array}$$ (4)

Eq (4) can be written as [22]

$$\begin{array}{c} \nabla^{2}P_{i}=\frac{\alpha^{2}}{R^{2}}\left(P_{i}-P_{ss}\right), \end{array}$$ (5)

where

$$\begin{array}{c} \alpha =R\sqrt{(L_{p}S+L_{pL}S_{L})/kV}, \end{array}$$ (6)

$$\begin{array}{c} P_{ss}=\left(L_{p}S/VP_{e}+L_{pL}S_{L}/VP_{L}\right)/\left(L_{p}S/V+L_{pL}S_{L}/V\right), \end{array}$$ (7)

and *R* is the tumor radius. The dimensionless parameter *α* measures the ratio of the vascular conductance to the interstitial conductance. *P*_*ss*_ is the steady-state pressure, a weighted average of *P*_*e*_ and *P*_*L*_ at which the flow rate outflux from blood vessels to the interstitium equals the influx from interstitium into lymphatic vessels. The effective pressure is defined as *P*_*e*_ = *P*_*B*_ − *σ*_*s*_(*π*_*B*_ − *π*_*i*_). Note that it seems *P*_*ss*_ in the tumor will be a function of *r*, since *S*/*V* and *S*_*L*_/*V* are generally functions of *r*, as depicted in Fig 2. However, with *S*/*V* and *S*_*L*_/*V* both being zero in the necrotic core and having sine distribution in other region of tumor, *P*_*ss*_ will be reduced to a piecewise constant distribution in tumor. Together with *S*/*V* and *S*_*L*_/*V* being constants in normal tissues, *P*_*ss*_ can be more specifically expressed as,

$$\begin{array}{c} P_{ss}=\{\begin{array}{cc} P_{ss1}=0, & 0\leq r\leq R_{n},\\ P_{ss2}=\frac{L_{p}{\left(S/V\right)}_{\text{max}}P_{e}+L_{pL}{\left(S_{L}/V\right)}_{\text{max}}P_{L}}{L_{p}{\left(S/V\right)}_{\text{max}}+L_{pL}{\left(S_{L}/V\right)}_{\text{max}}}, & R_{n}\leq r\leq R\\ P_{ss3}=\frac{L_{p}{\left(S/V\right)}_{\text{normal}}P_{e}+L_{pL}{\left(S_{L}/V\right)}_{\text{normal}}P_{L}}{L_{p}{\left(S/V\right)}_{\text{normal}}+L_{pL}{\left(S_{L}/V\right)}_{\text{normal}}}, & R<r. \end{array} \end{array}$$ (8)

Because of symmetry, a pole condition with the pressure gradient (hence the interstitial fluid flow velocity) being zero is implemented at the center of tumor:

$$\begin{array}{c} \frac{\partial P_{i}}{\partial r}=0\;\text{at}\;\;r=0. \end{array}$$ (9)

For a subcutaneous tumor, the boundary conditions at the tumor-normal tissue interface, based on the continuity of pressure and velocity, are as follows,

$$-k_{t}{\frac{dP_{i}}{dr}|}_{r=R^{-}}=-k_{n}{\frac{dP_{i}}{dr}|}_{r=R^{+}},$$ (10)

and

$${P_{i}|}_{r=R^{-}}={P_{i}|}_{r=R^{+}},$$ (11)

where *k*_*t*_ and *k*_*n*_ are the hydraulic conductivity of the interstitium in tumor and normal tissues. Another requested boundary condition for pressure in normal tissue is that

$$\begin{array}{c} P_{i}\to P_{ss3},\;r\to \infty , \end{array}$$ (12)

meaning the interstitial pressure would approach its *P*_*ss*_ in normal tissue when far from the tumor edge.

**Fig 3:**
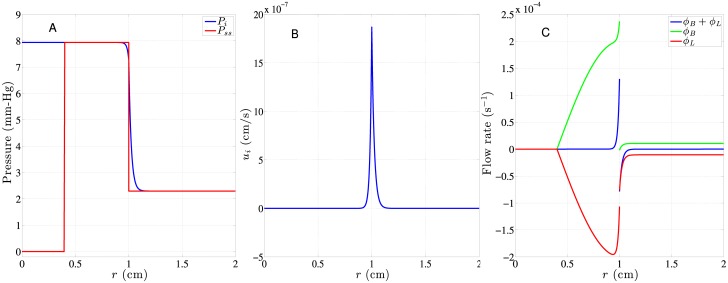
Hydrodynamic distributions based on the *S*/*V* and *S*_*L*_/*V* distributions in Fig 2A and parameters listed in Table 1. Distributions of A: *P*_*i*_, B: *u*_*i*_ and C: *ϕ*_*B*_ (according to Eq (2)), *ϕ*_*L*_ (according to Eq (3)), and their sum in a 2-cm subcutaneous (*R*_*n*_ = 0.4*R*) tumor.

All the equations above describe the hydrodynamics of interstitial fluid in a subcutaneous tumor. Eq (5) will be solved to obtain the interstitial pressure distribution *P*_*i*_. After that, the interstitial fluid flow velocity ${\vec{u}}_{i}$, flow source *ϕ*_*B*_, and flow sink *ϕ*_*L*_ would then be computed, which will be used in the drug transportation model described below. A typical *P*_*i*_ and *P*_*ss*_ distributions in a subcutaneous tumor would look like Fig 3A while the accompanied ${\vec{u}}_{i}$, and *ϕ*_*B*_, *ϕ*_*L*_ are shown in Fig 3B and 3C, respectively. The figure shows that the interstitial pressure *P*_*i*_ remains constant with the value being tumor’s *P*_*ss*2_ within most of the tumor, and has a steep descent across the interface of tumor and normal tissues, and then decays to another constant value of *P*_*ss*_ in normal tissues. This causes large outward interstitial fluid flow around the interface, as shown in Fig 3B, and the accompanied drug convection would tend to push drug away from tumor and towards normal tissues. It might degrade the therapeutic effect and be more harmful to normal tissues at the same time. One might wonder why most part of the tumor, especially in the non-vascular necrotic core, experiences high interstitial pressure *P*_*ss*2_. This is understandable that the fluid inside the necrotic core is trapped there and is forced to have the same pressure as *P*_*ss*2_. This is mainly because all the vascularized region is mostly in the periphery of tumor and this pressure source forms a large back pressure at the core of tumor due to lack of lymphatic vessels to release the pressure.

Because *S*/*V* and *S*_*L*_/*V* in tumor tissues share the same shape of sine distribution here, *P*_*ss*_ yields a piecewise constant distribution, as shown in Eq (8). Under this circumstance, the effects of radial distributions of *S*/*V* and *S*_*L*_/*V* on the interstitial pressure happen to be exactly the same as *S*/*V* and *S*_*L*_/*V* specified to be constant distributions throughout the tumor like in [13]. Although having the same interstitial pressure distribution, hence the same interstitial fluid velocity distribution, these two different distributions of *S*/*V* and *S*_*L*_/*V* in [13] and the current model still will have different influence on drug transport through Eqs (14) and (15).

### Drug transport

The flux of drug in the interstitium can be described as $\vec{N}=-D\nabla C_{i}+\vec{u}C_{i}$, where *C*_*i*_ is the interstitial drug concentration, *D* is the diffusion coefficient (cm^2^/sec), and −*D*∇*C*_*i*_ and $\vec{u}C_{i}$ denote the diffusion and convection terms, respectively. Diffusion is driven by the concentration gradient and convection is governed by the fluid velocity. Besides these two processes, the drug source from blood vessels, drug drainage by lymphatic vessels and the decay of drug concentration in the interstitium due to uptake of drug by tumor and normal cells immersed in interstitium are also taken into consideration. As a result, the drug delivery equation is expressed as

$$\begin{array}{c} \partial C_{i}/\partial t+\nabla \cdot \vec{N}=\varphi_{SB}+\varphi_{SL}-\varphi_{R}, \end{array}$$ (13)

where *φ*_*R*_ = *R*_*d*_ *C*_*i*_ represents the drug uptake with *R*_*d*_ (1/s) denoting the uptake rate or sink coefficient; *φ*_*SB*_ (mole/cm^3^-s) and *φ*_*SL*_(mole/cm^3^-s) are respectively the solute effluxes from blood vessels and lymphatic vessels per unit volume, which are described by Kedem-Katchalsky equation as

$$\begin{array}{c} \varphi_{SB}=\phi_{B}\left(1-\sigma_{s}\right){\bar{C}}_{s}+\omega S/V\Delta C, \end{array}$$ (14)

where *ω* is microvascular permeability (cm/s), Δ*C* = *C*_*h*_ − *C*_*l*_ (mole/cm^3^) and ${\bar{C}}_{s}=\frac{C_{h}-C_{l}}{\ln\left(C_{h}C_{l}^{-1}\right)}\approx \frac{1}{2}\left(C_{h}+C_{l}\right)$ are the concentration difference and average of solutions placed at both sides of the blood vessel membrane, respectively. Here the high concentration *C*_*h*_ will be the drug concentration in blood vessels *C*_*B*_ and the low concentration *C*_*l*_ will be *C*_*i*_. Likewise, the rate of solute transport across the lymphatic vessel *φ*_*SL*_ can also be expressed as

$$\begin{array}{c} \varphi_{SL}=\phi_{L}\left(1-\sigma_{L}\right){\bar{C}}_{L}+\omega_{L}S_{L}/V\Delta C_{L}, \end{array}$$ (15)

where *ω*_*L*_ is permeability for lymphatic vessels (cm/s), Δ*C*_*L*_ (mole/cm^3^) and ${\bar{C}}_{L}$(mole/cm^3^) are the concentration difference and average of solutions placed at both sides of the lymphatic vessel membrane, respectively. The expression of Δ*C*_*L*_ and ${\bar{C}}_{L}$ are same as Δ*C* and ${\bar{C}}_{s}$ above, but with *C*_*l*_ = *C*_*i*_ and *C*_*h*_ being the drug concentration in lymphatic vessels *C*_*L*_. *σ*_*L*_ is the average osmotic reflection coefficient for lymphatic vessels and is set to be the same as *σ*_*s*_ here. Similarly, the permeability of lymphatic vessels *ω*_*L*_ was set to be the same vascular permeability *ω* in the tissue. As to the boundary/interface conditions for Eq (13), at the center of the tumor, there is a pole condition

$${\frac{\partial C_{i}}{\partial r}|}_{r=0}=0.$$ (16)

At the interface of tumor and normal tissue, the concentration and its flux needs to be continuous over there:

$${C_{i}|}_{r=R^{-}}={C_{i}|}_{r=R^{+}}$$ (17)

and

$${\left(-D\frac{\partial C_{i}}{\partial r}+{\vec{u}}_{i}C_{i}\right)}_{r=R^{-}}={\left(-D\frac{\partial C_{i}}{\partial r}+{\vec{u}}_{i}C_{i}\right)}_{r=R^{+}},$$ (18)

where the latter can be further simplified to

$${\left(-D\frac{\partial C_{i}}{\partial r}\right)}_{r=R^{-}}={\left(-D\frac{\partial C_{i}}{\partial r}\right)}_{r=R^{+}},$$ (19)

by Eqs (10) and (17).

As *r* → ∞ in normal tissues, the boundary condition over there is a convective boundary condition, which is simply Eq (13) with the diffusion term neglected over there.

To close the equations, we still need governing equations for *C*_*B*_ and *C*_*L*_, which are governed by an exponential decay, characterized by the half-life decaying rate λ (1/s) and related source/sink terms from Eqs (14) and (15) based on mass conservation as

$$\begin{array}{c} \frac{dC_{B}}{dt}=-\lambda C_{B}-\varphi_{SB} \end{array}$$ (20)

and

$$\begin{array}{c} \frac{dC_{L}}{dt}=-\lambda C_{L}-\varphi_{SL}, \end{array}$$ (21)

where λ is related to the plasma half-life time *τ* as λ = ln 2/*τ*. Several things should be noted for Eqs (20) and (21). First, they are “local” compartment models. *C*_*B*_ and *C*_*L*_ are temporal-spatial variables like *C*_*i*_. They are consistent with the spatial distributions of *ϕ*_*B*_, *ϕ*_*L*_, *S*/*V* and *S*_*L*_/*V*, as shown in Eqs (14) and (15). However, due to circulation constraint to vessels, unlike Eq (13) there will be no diffusion and convection in them. This reduces Eqs (20) and (21) to ODE models. It means *C*_*B*_ and *C*_*L*_ can be computed locally without coupling to its neighborhood explicitly. Second, *C*_*B*_ and *C*_*L*_ have the same plasma half-life time λ here. It is because the drug decays in circulation system (consisted of both blood and lymphatic vessels) through the uptake of liver.

The initial conditions for *C*_*i*_, *C*_*B*_ and *C*_*L*_ are specified as

$$\begin{array}{c} C_{i}\left(r,0\right)=0, \end{array}$$ (22)

in both tumor and normal tissues and

$$\begin{array}{c} C_{B}\left(0\right)=C_{L}\left(0\right)=\{\begin{array}{c} C_{\text{max}},\;r\geq R_{n}\\ 0,\;r<R_{n} \end{array}. \end{array}$$ (23)

The equality of *C*_*B*_ and *C*_*L*_ initially is due to drug goes directly from blood vessels to lymphatic vessels at the instant of drug injection (not yet drained to the interstitium). The vanishing of *C*_*B*_ and *C*_*L*_ in the necrotic region is due to the lack of vasculature. Eqs (13), (20) and (21) will be solved for *C*_*i*_(*r*, *t*), *C*_*B*_(*t*) and *C*_*L*_(*t*) in both tumor and normal tissues with boundary/interface and initial conditions described above. Note that since *C*_*i*_(*r*, *t*), *C*_*B*_(*t*) and *C*_*L*_(*t*), are proportional to *C*_max_, judging from Eqs (13), (20) and (21). We can scale these concentrations by *C*_max_, which is equivalent to set *C*_max_ = 1 all the time.

### Parameter values

The material parameters of the tumor and normal tissues used in this work adopted the values published by Baxter and Jain [22], and these values are listed in Table 1. Note that *S*/*V* in tumor and normal tissues in [22] were in uniform distribution with constant value of 200 cm^-1^ and 70 cm^-1^ respectively. Since in our current model the *S*/*V* and *S*_*L*_/*V* are heterogeneously distributed in tumor, we set the maximum values of *S*/*V* to be 200 cm^-1^ [2, 5] and maximum values of *S*_*L*_/*V* to be 20 cm^-1^ in tumor tissues, and both to be uniformly distributed in normal tissues with a constant value of 70 cm^-1^. As no value of hydraulic conductivity for tumors has been reported in the literature [22], *L*_*pL*_ value can only be deduced from the reported lymphatic filtration coefficient *L*_*pL*_ *S*_*L*_/*V* and the estimated *S*_*L*_/*V* value [26]. *S*/*V* and *S*_*L*_/*V* have the same constant value in normal tissue, but different maximum values in tumor because normal tissue, compared with tumor, has a much more extensively functional lymphatic network, which removes the net fluid filtered from the blood vasculature. Thus the extravasated materials are more quickly re-absorbed by lymphatic vessels in normal tissues [22, 27]. Also, unlisted in Table 1, the pressure in lymphatic vessels *P*_*L*_ is 5 mmHg smaller than the effective pressure *P*_*e*_ according to [22].

**Table 1:** Hydrodynamic parameter values for tumor tissues of isolated and subcutaneous tumors as well as for normal tissues surrounding a subcutaneous tumor [22, 32].

Parameter	Tumor	Normal tissue
*L*_*p*_ (cm/mmHg-sec)	2.8 × 10^−7^	0.36 × 10^−7^
*L*_*pL*_ (cm/mmHg-sec)	6.94 × 10^−6^	1.90 × 10^−7^ [26]
*k* (cm^2^/mmHg-sec)	4.13 × 10^−8^	8.53 × 10^−9^
*S*/*V* (1/cm)	200*	70
*S*_*L*_/*V* (1/cm)	20*	70
*P*_*B*_ (mmHg)	15.6	15.6
*π*_*B*_ (mmHg)	20	20
*π*_*i*_ (mmHg)	15	10
*σ*_*s*_	0.82	0.91

*The maximum value for vascular distributions, as shown in Fig 2.

Dextrans with the molecular weight of 10 kDa, 70 kDa, and 2 MDa were chosen as the nanoparticle carriers and the characteristics of dextrans in a tumor are shown in Table 2 [28]. Nugent and Jain (1984) showed the diffusion coefficients for dextrans as a function of molecular weight according to the expression *D* = *a*(*M*)^*b*^ (cm^2^/sec), where the values for *a* and *b* are different in tumor and normal tissues [29]. The diffusivity values of 10 kDa, 70 kDa, and 2 MDa dextrans in tumor and normal tissues were estimated from Fig 4A of [30] and Fig 6 of [31] by extrapolation, interpolation, and extrapolation, respectively.

**Table 2:** Parameters of dextrans of different molecular weights in tumors.

Parameter	10 kDa dextran	70 kDa dextran	2 MDa dextran	Reference
Interstitial diffusivity (cm^2^/s)	1.72 × 10^−6^	1.4 × 10^−7^	5.31 × 10^−10^	[30, 31]
Vascular permeability *ω* (cm/s)	3.2 × 10^−6^	9.8 × 10^−7^	1.7 × 10^−7^	[31]
Sink (1/s)	1.00 × 10^−3^	4.17 × 10^−4^	1.23 × 10^−4^	[28]
Half life (min)	8.17	23.77	35.14	[28]

The vascular permeability of nanoparticles in the tumor was estimated to be about 7.8 times that of normal tissues, as measured by Gerlowski and Jain [31]. The counterpart for normal tissues are shown in Table 3. The parameter sink in Tables 2 and 3 is *R*_*d*_ in Eq (13), corresponding to the drug uptake in tissues. For comparison with the nanoparticle dextran, doxorubicin was chosen as the conventional molecular anti-cancer drug. The molecular weight of doxorubicin is 544 Da and Table 4 lists its transport parameters utilized in this work. Note that the vascular permeability *ω* generally has larger value in tumor than in normal tissue. For the reason that drug leaks to both tumor and normal tissues through blood vessels, but blood vessels in tumor are usually much more leaky than those in normal tissues due to the irregularly developed vasculature during angiogenesis.

**Table 3:** Parameters of dextrans of different molecular weights in normal tissues.

Dextran parameters	10 kDa	70 kDa	2 MDa	Reference
Interstitial diffusivity (cm^2^/s)	1.64 × 10^−6^	5.0 × 10^−9^	3.37 × 10^−13^	[30, 31]
Vascular permeability *ω* (cm/s)	4.06 × 10^−7^	1.24 × 10^−7^	2.16 × 10^−8^	[31]
Sink (1/s)	5.00 × 10^−4^	2.085 × 10^−4^	6.15 × 10^−5^	derived from [28]
Half life (min)	8.17	23.77	35.14	[28]

**Table 4:** Parameters of doxorubicin.

Parameter	Tumor	Normal tissue	Reference
Interstitial diffusivity (cm^2^/s)	3.40 × 10^−6^	1.58 × 10^−6^	[33]
Vascular permeability *ω* (cm/s)	3.00 × 10^−4^	3.75 × 10^−5^	[33]
Sink (1/s)	2.689 × 10^−3^	2.689 × 10^−3^	[34]
Half life (min)	5.3	5.3	[35]

### Tumor cell survival

The cumulative drug concentration can be computed by use of the equations above, which was subsequently used to estimate the cell survival rate of tumor and normal tissues after treatments. The therapeutic effect and the probability of tumor recurrence can be measured based on the tumor cell survival rate. In 1983, Greene et *al*. reported that the survival rate in a tumor was an exponential function of the extracellular concentration of anti-cancer drug [36]. In 2000, El-Kareh and Secomb argued that it would be more reliable to estimate the cell survival rate with the intracellular concentration in cells [12]. In our model, the cell survival rate (*S*_*F*_) was defined as *S*_*F*_ = *N*/*N*_0_, where *N*_0_ and *N* denote the numbers of cells before and after a treatment. The relationship between the cell survival fraction and the extracellular concentration was defined as:

$$\begin{array}{c} \ln S_{F}=-k_{s}\cdot \int_{0}^{\infty }C_{i}\left(r,t\right)dt=-k_{s}\;\text{AUC}\left(r\right), \end{array}$$ (24)

where *k*_*s*_ is the dose-survival constant and AUC, abbreviation for area under the curve, is a frequently used pharmacokinetic term denoting the cumulative concentration in the interstitium over time; in other words, the area under the concentration-time curve, which can be described as

$$\begin{array}{c} \text{AUC}\left(r\right)=\int_{0}^{\infty }C_{i}\left(r,t\right)dt. \end{array}$$ (25)

The dose-survival constant (*k*_*s*_) employed the value given in Jusko’s work [37], which is 4.329 × 10^−3^ (1/nM-hr). The cell survival rate after each single treatment can be estimated by use of Eq (24). In the field of pharmacokinetics, the area under the curve (AUC) is the definite integral (from zero to infinity) in a plot of drug concentration in tissue vs. time. The AUC represents the total drug exposure over time.

When estimating the cell survival rate after multiple treatments, the regrowth of tumor cells after each treatment needs to be considered. The tumor cell growth can be estimated by the three-parameter Gompertzian function [18, 19, 38]:

$$\begin{array}{c} n_{i}\left(t\right)=N_{i}\exp\{\ln\left(\frac{N_{\infty }}{N_{i}}\right)\left[1-\exp\left(-bt\right)\right]\}, \end{array}$$ (26)

where *N*_*i*_ is the number of viable tumor cells after *i*-th treatment; *n*_*i*_(*t*) is the number of tumor cells at time *t* after *i*-th treatment; *N*_∞_ is the saturated cell number that can be reached after a long period of time and *b* is related to the initial tumor growth rate [38]. Here we adopted the initial number of tumor cells as *N*_0_ ≈ 5 × 10^9^, *N*_∞_ = 3.1 × 10^12^ and *b* = 0.0283 month^-1^ from the work of Yorke et *al*. [38]. The cell number for the 1-cm thick normal tissue was assumed *N*_∞_ = 4.64 × 10^12^ here. We assumed that the number of cells is proportional to the tumor volume, which means that the tumor size shrinks (*R* decreases) as the number of cells decreases, and assumed that the ratio of normal tissue density to tumor cell density to be 0.2 [39]. Another assumption is that the shape of vascular density distribution in the tumor remains unchanged after each treatment. Moreover, the regrowth of normal tissue cells was also evaluated by use of Eq (26) under the assumptions that the mean growth rate *b* of normal tissues is half of that in a tumor.

**Fig 4:**
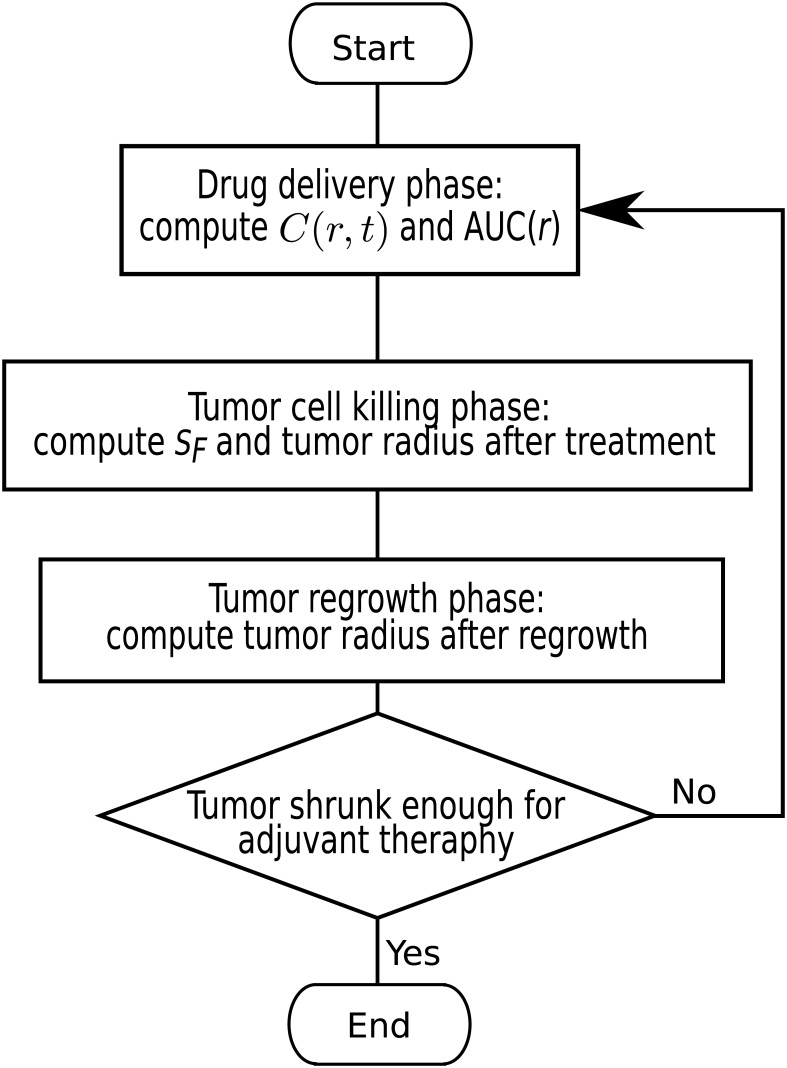
The flowchart that descries the repeated procedures in multiple treatments.

The whole simulation procedures are summarized and depicted by the flowchart contained in Fig 4. The flow chart describes the repeated procedures in multiple treatments consisted of the following stages: (1) drug delivery phase: computing *C*(*r*, *t*) and AUC(*r*), (2) tumor cell killing phase: computing cell survival rate *S*_*F*_ and tumor radius after treatment, (3) tumor regrowth phase: computing tumor radius after regrowth.

### Numerical method

Here we employ method of lines (MOL) with the semi-discretization in space at first for Eq (13) by multi-block Chebyshev pseudospectral method. Together with interface/boundary conditions, it then would result in a set of ordinary differential algebraic equations (ODAEs) in time. We can then solve this ODAE set by a highly efficient variable-step-variable-order (VSVO) ODAE solver like ode15s in Matlab. Due to the high order of accuracy intrinsic to pseudospectral method, far less grid points are needed to achieve quite accurate numerical results compared with traditional finite difference/element methods. Also, since ode15s has an error estimate at each step, it can automatically adjust its time step to avoid instability and gain the optimal efficiency in time integration.

## Results and discussions

Our model can compute the spatial distribution of the interstitial pressure and the cumulative drug concentration featured by AUC. Furthermore, the spatially-averaged AUCs resulted from different anti-cancer agents can be calculated and hence the therapeutic effect of nanoparticle carriers and molecular chemotherapeutic agents can be evaluated via the cell survival rate. A 2-cm (diameter) subcutaneous tumor with a 0.8-cm necrotic region, i.e., *R* = 1 cm and *R*_*n*_ = 0.4*R*, embedded in a 1 cm thick normal tissues was used in our current study.

**Fig 5:**
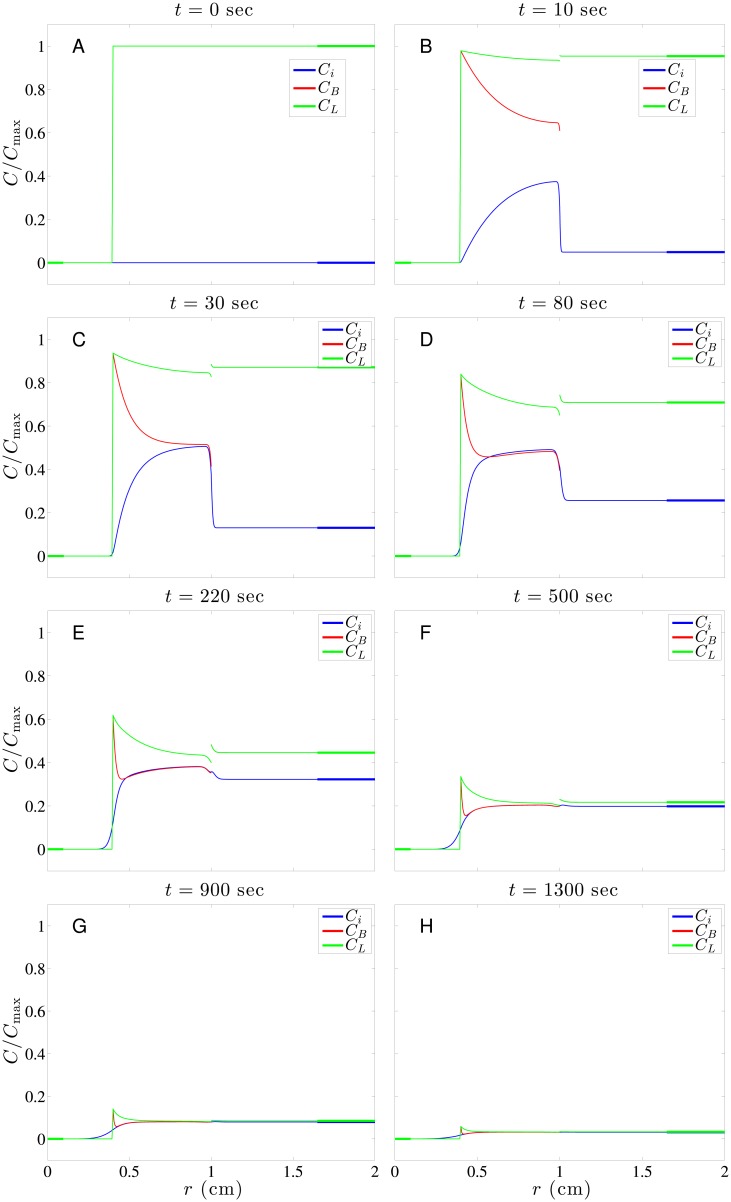
A series of snapshots showing time evolution of doxorubicin concentration distribution in a subcutaneous tumor. A: *t* = 0; B: *t* = 10, C: *t* = 30, D: *t* = 80, E: *t* = 220, F: *t* = 500, G: *t* = 900; H: *t* = 1300 sec.

Fig 5 shows the typical time evolution of drug concentration distributions in a subcutaneous tumor. This time evolution shows different behaviors in the tumor necrotic region, tumor viable region and normal tissue surrounding the tumor. In the necrotic region, since neither blood nor lymphatic vessels are present and the interstitial pressure is flat there as well, the convection and source/sink terms vanish in Eq (13). The only way for drug to penetrate the necrotic region is through diffusion, which is generally weak there. Though most tumor cells are already deceased in the necrotic region, it does not mean the amount of the drug penetration is insignificant in treating a tumor since there are still some surviving tumor stem cells hiding inside. In the viable tumor region, the drug level increases fast in the beginning due to double sources from the permeation terms of *φ*_*SB*_ and *φ*_*SL*_ in Eq (13). The first term in Eq (14) acts as the source while the first term in Eq (15) acts as sink due to *ϕ*_*B*_ and *ϕ*_*L*_, as shown in Fig 3C. These two terms roughly cancel each other except around the interface of tumor and normal tissues. The second term in Eqs (14) and (15) is the major source term for drug transported from vessels (blood and lymphatic vessels) into the interstitium, and the driving force is the concentration difference between vessels and interstitium. This driving force is modulated by the vessel permeability (*ω* and *ω*_*L*_) and vascular density (*S*/*V* and *S*_*L*_/*V*). Also the increase of *C*_*i*_ is biased towards the interface due to convection, as shown in Fig 3B. Accompanying the increase of *C*_*i*_ naturally comes the decay of both *C*_*B*_ and *C*_*L*_. Here *C*_*B*_ decays much faster than *C*_*L*_ in the viable tumor region chiefly due to *S*/*V* >> *S*_*L*_/*V*.

In normal tissues, unlike the viable tumor region, the interstitial concentration *C*_*i*_ increases very slowly in the beginning due to small values of *φ*_*SB*_ and *φ*_*SL*_. This is a result of the much smaller vascular permeability (*ω* and *ω*_*L*_) in normal tissues compared with its counterpart in tumors, as shown in Table 4. In addition, smaller vascular densities (*S*/*V* and *S*_*L*_/*V*) in normal tissue than those in tumor contribute to this slow increase as well. Drug penetrating into normal tissue through convection (driven by a higher interstitial pressure in a tumor) and diffusion (due to faster growth of drug concentration in the viable tumor region in the beginning) also causes the increase of drug concentration in normal tissue, but only limited to normal tissues near the interface. Generally, we wish AUC for normal tissues to be as small as possible to avoid harming normal tissues. As time goes, *C*_*i*_ changes from increasing to decreasing and dies out eventually due to continuous weakening of driving force and the uptake of drug by cells in interstitium.

**Fig 6:**
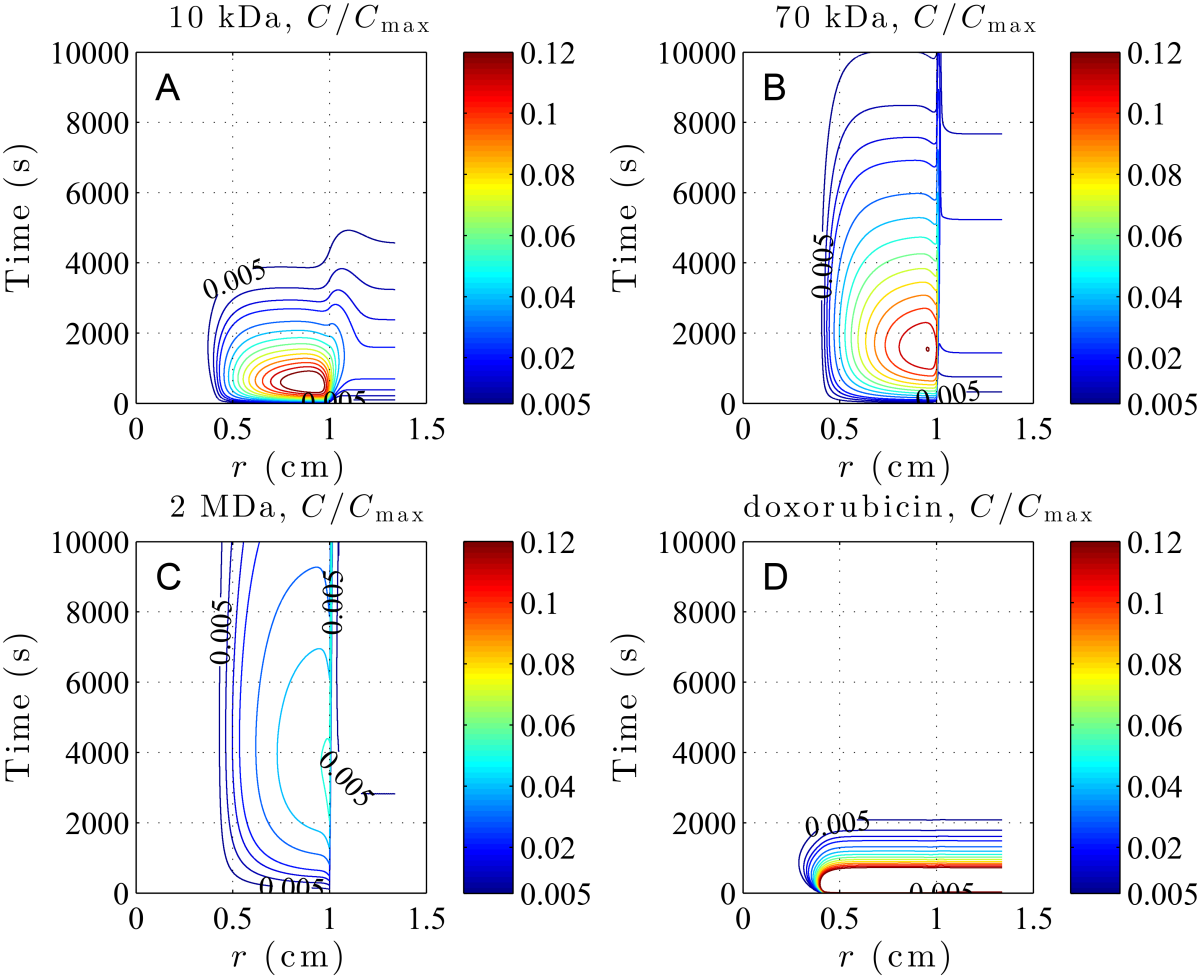
The concentration contours showing time evolution of dextrans and doxorubicin distributions in a subcutaneous tumor. A: 10 kDa dextran, B: 70 kDa dextran, C: 2 MDa dextran and D: doxorubicin. The smallest contour value *C*/*C*_max_ = 0.005 is marked.

**Fig 7:**
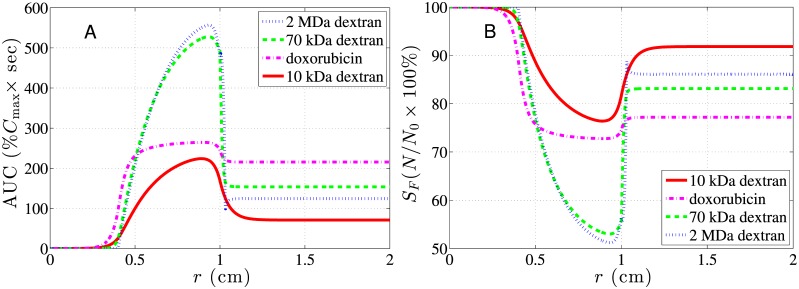
Spatial distributions of AUC and *S*_*F*_ for dextrans and doxorubicin in a subcutaneous tumor. A: The spatial distribution of 10-kDa, 70-kDa, 2-MDa dextrans and doxorubicin. B: The corresponding spatial distribution of the cell survival rates. The 2-cm (*R* = 1 cm) tumor with a 0.8-cm (*R*_*n*_ = 0.4*R*) necrotic region was immersed in a 1 cm thick normal tissues.

The concentration contours of 10 kDa, 70 kDa, and 2 MDa dextrans as well as that of doxorubicin are displayed in subfigures A-D of Fig 6. It can be seen from Fig 6A and 6D that concentrations of small particles like 10 kDa dextran and molecular drug doxorubicin have larger contour values, but decay quickly over time. On the contrary, larger nanoparticles like 70 kDa and 2 MDa dextrans have small contour values, but decay much more slowly. This is mainly controlled by vascular permeability. As shown in Tables 2 and 4, vascular permeability monotonically decreases with molecular weight. Generally speaking, a larger vascular permeability causes drug to leak more quickly from vessels into interstitium, result in a higher level of interstitial concentration, but at the same time decay more quickly. On the contrary, smaller vascular permeability causes drug to leak slowly from vessels into interstitium, result in smaller levels of concentration, but sustain longer before vanishing. Both vascular permeability and density are smaller in normal tissue than in tumor; consequently, less drug is able to leak from vessels into the interstitium. As a result, drug concentration levels are generally lower in normal tissue than in tumor. Note that the concentration contours of 2 MDa dextran exist almost strictly in the viable tumor region, which indicates almost no drug transport from tumor into normal tissue. This can be attributed to its small concentration difference between tumor and normal tissues owing to the smallest vascular permeability among all drugs. Besides, its smallest diffusion coefficients among all drugs also hinders the diffusion of drug from tumor to normal tissues. It naturally implies that 2 MDa dextran kills tumor cells most and at the same time harms normal tissue least. Actually, the therapeutic effect of drug should be evaluated by its AUC distribution, i.e., the time integral of the local concentration. Fig 6 shows the contours of drug concentration *C*_*i*_(*r*, *t*) computed from Eq (13), and its time integral from zero to infinity will generate the spatial distribution of AUC like Fig 7A. Therefore, both the drug concentration level and its duration in the interstitium are important contributing factors to AUC. Judging from 2 MDa and 70 kDa dextrans having larger spatial distributions of AUC in tumor tissues than 10 kDa dextran and doxorubicin, as shown in Fig 7A, it appears that the duration time affects the AUC more significantly than the momentary concentration levels here.

The spatial distributions of AUC are shown in Fig 7A and the average values in different regions are listed in Table 5. This gives us a more precise therapeutic effect prediction than Fig 6. All anti-cancer drugs shared a similar distribution pattern in AUC. Compared with the normal tissue region, their AUCs in the tumor vascular region were usually high, but extremely low in the necrotic core. This low AUC in the necrotic region is simply because there is no vascular vessel there to transport drugs. Therefore, very limited amount of drug is transported into a necrotic region solely via diffusion from a vascular region. Also, this low AUC in the necrotic region might imply insufficient dosage to kill the tumor stem cells hiding in it. Note that smaller drugs penetrate deeper into normal tissues and affect more normal tissues while larger nanoparticles display a shallower penetration. This has been analyzed via Fig 6. The survival rate of normal tissue will be particularly affected by the degree of penetration.

**Table 5:** The spatially-average AUCs in the tumor using various sized dextrans and doxorubicin.

Tissue type	${\bar{\text{AUC}}}_{10\text{kDa}}^{*}$	${\bar{\text{AUC}}}_{70\text{kDa}}^{*}$	${\bar{\text{AUC}}}_{2\text{MDa}}^{*}$	${\bar{\text{AUC}}}_{\text{DOX}}^{*}$
Vascular region	190.19	447.50	438.35	254.71
Necrotic region	6.62	2.76	0.27	21.12
Normal tissue	73.40	155.39	100.40	215.74

*The unit of AUC is %*C*_max_ × sec, where *C*_max_ is the injected drug dose.

As shown in Table 5, the average AUCs of larger-sized dextrans were much higher in the vascular region and lower in normal tissue than those of doxorubicin, which suggests that larger-sized dextrans generally have a better therapeutic effect and less side effect than doxorubicin. This shows that using larger-sized nanoparticles as drug carriers is more effective at treating a tumor and less harmful to the surrounding normal tissues than molecular chemotherapeutic agents. Based on average AUCs, 2 MDa dextran has the best therapeutic effect to the vascular tumor region and second least side effect to normal tissues, and this generally indicates that larger nanoparticles are the better choice for treating a well-vascularized tumor.

Besides calculating the average AUC of drugs, we estimated the cell survival rate based on Eq (24) and used it to quantify the therapeutic effect after treatments, as shown in Fig 7B. Consistently, 2 MDa dextran has the best performance with lowest survival rate distribution in tumor and second highest survival rate distribution in normal tissue.

**Fig 8:**
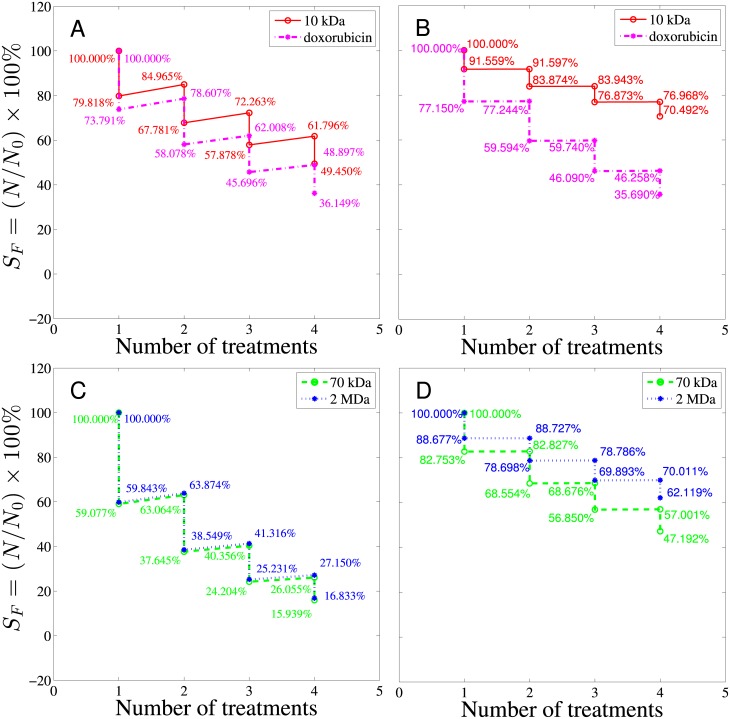
The overall cell survival rate for tumor and normal tissues after four treatments. A and C show the survival rates in tumor while B and D show the survival rates of normal tissues after 4 treatments of drugs. The abscissa represents the number of treatments with 10-day treatment interval. *C*_max_ is the maximum concentration in the blood vessel, which is taken to be 1000 nM here.

The tumor cell responses to all four kinds of drug after multiple treatments were also investigated and the spatially-averaged survival rates of viable tumor region and normal tissue are depicted in Fig 8. The survival rate drops after each treatment followed by regrowth based on Eq (26) for both tumor and normal tissue. For comparison, in Fig 8, doxorubicin and 10 kDa are grouped together to represent smaller-sized drugs while 70 kDa and 2 MDa dextrans are grouped together to represent larger-sized drugs. As shown by the red curve in Fig 8A, the tumor cell survival rate after the first treatment of 10 kDa dextran was about 79.82% and about 50.55% of the tumor cells were killed after the fourth treatment. The tumor cell survival rate after each treatment of doxorubicin is depicted by the magenta, dashed curve in Fig 8A, which was about 73.79% after one doxorubicin treatment. The overall tumor cell survival rate was about 36.15% after four doxorubicin treatments. In addition to the effectiveness of killing cancer cells, the side effects to normal tissues during chemotherapy also needs to be addressed. Therefore, the cell survival rates of normal tissue were also investigated. From Fig 8B, the normal tissue survival rate after the first treatment of 10 kDa dextran delineated by the red curve was about 91.56%, and about 70.49% of normal tissue cells were left after the whole therapy program. At the same time a large fraction of normal tissue was killed by doxorubicin treatment (the survival rate after the first treatment was about 77.15%) and only 35.69% of normal tissue cells were left after the whole therapy program. It is worth noting that the normal tissue survival rate was almost the same as the tumor survival rate in doxorubicin treatment. In other words, doxorubicin kills cancer and normal cells without distinguishment. These results show that 10 kDa dextran, though less efficient at killing tumor cells, was less harmful to normal tissues compared with doxorubicin. Fig 8C and 8D show the tumor and normal tissue survival rates after multiple treatments of 70 kDa and 2 MDa dextrans. For multiple treatments, larger-sized drugs like 70 kDa and 2 MDa dextrans are generally more effective in killing tumor cells compared with smaller-sized drugs like doxorubicin and 10 kDa dextran, and have a normal tissue survival rate between doxorubicin and 10 kDa dextran, which can be comprehended by AUC and *S*_*F*_ in the normal tissue region shown in Fig 7.

**Fig 9:**
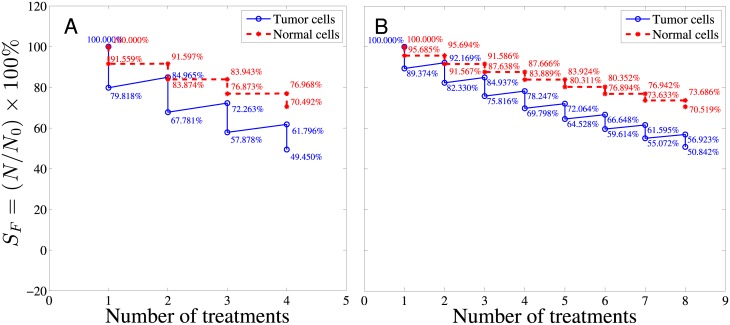
The overall survival rate after 10 kDa dextran treatments. The survival rates of tumor cells (blue curve) and normal cells (red dashed curve). A: The maximum concentration (*C*_max_) in the blood vessel was assumed to be 1000 nM and four treatments were administered with a time interval between two treatments of 10 days. B: The maximum concentration (*C*_max_) in the blood vessel was assumed to be 500 nM and the total number of treatments was eight times with a time interval between two treatments of 5 days.

Effects of different treatment plans can be compared by the current model as well, as depicted by Fig 9. In the first therapy program, the maximum concentration (*C*_max_) in the blood vessel was assumed to be 1000 nM and the total number of treatments was four times with 10 days interval between two treatments. In the second therapy program, the maximum concentration (*C*_max_) in the blood vessel was cut into half to be 500 nM and 8 treatments were administered with 5 days interval between two treatments. The tumor and normal tissue cell survival rates resulted from these two programs were delineated in Fig 9. The result shows that the performance differences between 4 longer interval treatments and 8 shorter interval treatments are negligible, judging from both the tumor and normal tissue survival rates. Though the dose per treatment in the second plan is half of the first one and seems to be less toxic to the patient, it does not end up with a higher survival rate of normal tissue than the first treatment. The figure of the tumor and normal tissue cell survival rates after multiple treatments provides important prognostic information regarding the survival rate and percentage of the cell regrowth. It is of great help to evaluate the efficiency of a treatment plan. Note that in these multiple-treatment simulations, *S*/*V* and *S*_*L*_/*V* have been fixed values as tabulated in Table 1. However, angiogenesis after each treatment may actually be different and heterogeneity in the spatial distribution of blood and lymphatic vessels may occur, which means *S*/*V* and *S*_*L*_/*V* may vary after each treatment. Not just angiogenesis, even geometry of tumor would be different after each treatment. All these effects are not considered here, but will be studied in the future work by extending the current model to higher dimensions.

## Conclusions

The proposed model was employed to compute the interstitial pressure, dynamics of drug transport, AUC distributions of drugs of various sizes, and tumor/normal tissue cell survival rates in a subcutaneous tumor. The model is useful to quantify the therapeutic effect. It has been found that AUCs of all drugs are generally high in the tumor vascular region, low in normal tissues, and extremely low in the necrotic region. Larger nanoparticles like 2 MDa and 70 kDa dextrans display high AUCs in the tumor vascular region and, on the contrary, smaller nanoparticle drugs like 10 kDa dextran and molecular anti-cancer agents like doxorubicin show low AUC there. AUC shows that the delivery of all kinds of anti-cancer agents in the necrotic region was insufficient to kill the tumor stem cells hiding in it. Therefore, other treatment methods such as surgical removal and thermal therapy may be used to enhance the effectiveness of cancer treatment. The distribution of cell survival rates demonstrated that the side effect to normal tissues by use of dextrans was limited to a small range while doxorubicin caused damage extensively. Overall, larger-sized nanoparticles were found to deliver better therapeutic effects to the tumor region with limited toxicity to the surrounding tissues, as compared with the molecular anti-cancer agents such as doxorubicin.

In addition to estimating the delivery of drugs in tumors, the current model can be of help in the treatment planning. By estimating the cell survival rate after each treatment and regrowth between treatments, our method can be used to compare treatment plans with different parameters like dosage and frequency of treatment cycle.

## Future works

In the future, we plan to extend the present model to a more practical and complete one by the modification of the following three modules. (1) Geometry and hydrodynamics module: considering tumor with a geometry of higher dimensions; outlining explicit distributions of leaky blood and lymphatic vessels through angiogenesis into the model while still treating tumor interstitium as a porous media and modeling it by Darcy’s law. (2) Drug transport module: modifying the current drug transport model for the tumor environment mentioned in (1). (3) Time evolution of the spatial distribution of tumor cell density module: deriving a set of differential equations describing the rate of tumor cell density changes at least by its growth with nutrition supplied, death caused by the drug uptake in tumor, and natural death. By considering the above modified modules, simulations of this new model will shed more light on significant mechanisms of drug delivery inside tumor and, at the same time, offer a better and more practical evaluation of tumor treatment.
